# Targeting muscle stem cell intrinsic defects to treat Duchenne muscular dystrophy

**DOI:** 10.1038/npjregenmed.2016.6

**Published:** 2016-06-09

**Authors:** Nicolas A Dumont, Michael A Rudnicki

**Affiliations:** 1 Sprott Center for Stem Cell Research, Ottawa Hospital Research Institute, Regenerative Medicine Program, Ottawa, ON, Canada; 2 Department of Cellular and Molecular Medicine, Faculty of Medicine, University of Ottawa, Ottawa, ON, Canada

## Abstract

Duchenne muscular dystrophy (DMD) is a genetic disease characterised by skeletal muscle degeneration and progressive muscle wasting, which is caused by loss-of-function mutations in the *DMD* gene that encodes for the protein dystrophin. Dystrophin has critical roles in myofiber stability and integrity by connecting the actin cytoskeleton to the extracellular matrix. Absence of dystrophin leads to myofiber fragility and contributes to skeletal muscle degeneration in DMD patients, however, accumulating evidence also indicate that muscle stem cells (also known as satellite cells) are defective in dystrophic muscles, which leads to impaired muscle regeneration. Our recent work demonstrated that dystrophin is expressed in activated satellite cells, where it regulates the establishment of satellite cell polarity and asymmetric cell division. These findings indicate that dystrophin-deficient satellite cells have intrinsic dysfunctions that contribute to muscle wasting and progression of the disease. This discovery suggests that satellite cells could be targeted to treat DMD. Here we discuss how these new findings affect regenerative therapies for muscular dystrophies. Therapies targeting satellite cells hold great potential and could have long-term efficiency owing to the high self-renewal ability of these cells.

## Introduction

Duchenne muscular dystrophy (DMD) is an X-linked recessive disease that affects ~1 in 3,600 boys that is characterised by progressive debilitating muscle weakness resulting in gradual ambulatory disability, respiratory dysfunction and ultimately premature death in the second to third decade of life.^1^ DMD is caused by mutation in the *DMD* gene, which is the largest gene of the human genome that encompasses ~2.2 Mb and encodes for the dystrophin protein.^2,3^ In skeletal muscle, full-length dystrophin is expressed in myofibers where it binds to the actin cytoskeleton with its N-terminal domain and to the dystrophin-associated glycoprotein complex (DGC) at the cell membrane level with its C-terminal domain. In absence of dystrophin, myofibers are unstable and fragile, which causes progressive skeletal muscle degeneration.

Skeletal muscle contains muscle stem cells, named satellite cells, that are the engine of muscle regeneration.^4^ In healthy condition, satellite cells possess a tremendous capacity to regenerate muscles with their ability to proliferate extensively, differentiate and self-renew.^5^ However, in dystrophic muscles, the proliferating capacity of satellite cell is reduced and the overall muscle regeneration is impaired.^6–12^ Satellite cell exhaustion have been suggested to contribute to the reduced regenerative ability.^13^ However, although a decrease in the number of satellite cells is observed during aging of *mdx* or DMD muscles, that number remains equal or higher in dystrophic muscles compared to aged-matched healthy muscles.^6,14,15^

Pioneer work showed that deletion of *Dag1*, the transmembrane laminin-binding protein of the DGC, specifically in the myofibers induces a relatively mild degeneration phenotype compared with complete deletion of *Dag1*, i.e., in both the myofibers and the satellite cells.^16^ The authors suggested that Dag1 expression in satellite cells is essential to maintain their regenerative potential and, consequently, inadequate muscle repair caused by Dag1-deficiency in satellite cells is an important mechanism affecting the severity of muscular dystrophy.^16^ Similar results were obtained by analysing muscle regeneration of mice deficient in *fukutin*, a protein regulating Dag glycosylation and function.^17^ Conditional deletion of *fukutin* specifically in myofibers using muscle creatine kinase promoter also leads to a mild muscle phenotype compared with the severe muscle wasting observed when *fukutin* is specifically deleted in muscle precursor cells using the Myf5 promoter.^17^

**Figure 1:**
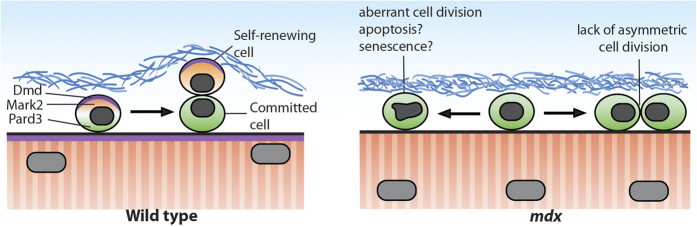
Dystrophin regulation of asymmetric cell division. Schematic micrograph of wild type (left panel) and *mdx* (right panel) dividing satellite cells. In wild-type mice, activated satellite cells express dystrophin that acts as a scaffolding protein for the cell polarity effector Mark2. Mark2 interacts with Pard3 leading to their asymmetric distribution and to asymmetric cell division. Absence of dystrophin in *mdx* mice leads to defect in cell polarity establishment and lack of asymmetric cell division. Impaired cell polarity in dystrophin-deficient satellite cell also increases the proportion of abnormal cell division.

Our recent work demonstrates that dystrophin (as well as other members of the DGC) is expressed in activated satellite cells where it regulates satellite cell fate and myogenesis.^18^ Dystrophin and Dag1 are expressed in a subset of activated satellite cells and are asymmetrically polarised prior to the first cell division. In activated satellite cells, dystrophin and Dag1 act as scaffolding proteins to which binds the cell polarity effector Mark2 (also known as Par1b). Dystrophin/Dag1-Mark2 interaction promotes the phosphorylation of the cell polarity regulator Pard3 leading to its asymmetric segregation at the opposite pole of the cell (Figure 1). Asymmetric cell polarity establishment leads to orientation of the mitotic spindle in an apicobasal orientation, which give rise to asymmetric cell division. Asymmetric cell division is a hallmark of stem cells that enables them to generates two cells with different cellular fates, one that remains a stem cell and the other one that becomes a committed progenitor cell. In skeletal muscle, asymmetric cell division enables muscle stem cells to maintain the satellite cell reserve (self-renewal) and simultaneously to contribute to the myogenic progenitor population that is needed for myofiber regeneration. On the other hand, muscle stem cells can also perform symmetric division to expand the stem cell pool.^19^ A controlled balanced between symmetric and asymmetric division is crucial to appropriately fulfill the needs of the muscles.

In absence of dystrophin, the cellular polarity of satellite cells is perturbed leading to the loss of asymmetric cell division and to an increase in the number of abnormal cell division. Lack of asymmetric cell division leads to a reduction in the number of myogenic progenitor cells and to impaired muscle regeneration (Figure 1).^18,20^ These findings indicate that there is a cell-autonomous defect in *DMD*-mutant satellite cells, which contributes to the pathogenesis of DMD. Understanding the molecular mechanisms driving satellite cell dysfunctions in dystrophic muscles opens new therapeutic avenues for the treatment of DMD. Here we discuss the consequences of these findings on the therapeutic strategies targeting satellite cells as well as potential implications for other diseases.

## DGC regulation of cell signalling

**Figure 2:**
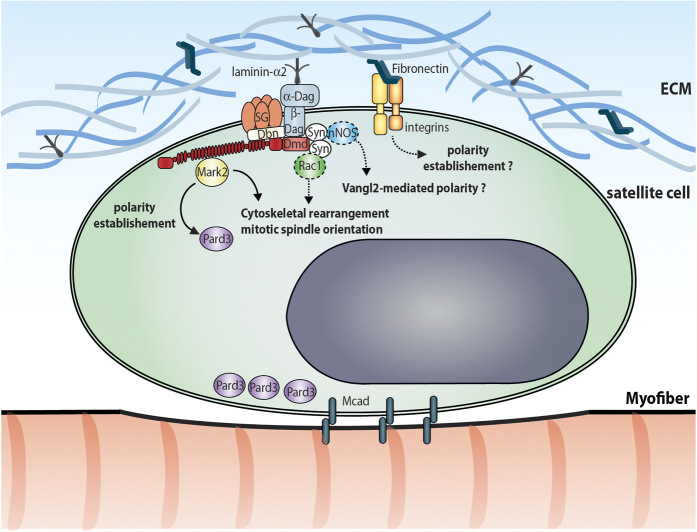
DGC regulation of satellite cell polarity establishment. Schematic micrograph of a satellite cell in its niche. DGC and integrins interacts with components of the extracellular matrix leading to their asymmetric distribution on the basal lamina side. Dystrophin and Dag1 interact with Mark2 to establish cell polarity. In myofibers, other DGC components were shown to interact with effectors involved in cell polarity establishment such as Rac1 and nNOS. Whether similar interaction occurs in satellite cells remains to be determined (dashed line). Dag, dystroglycan; Dbn; dystrobrevin; Dmd, dystrophin; Mcad, M-cadherin; SG, sarcoglycan; Syn; syntrophin.

In myofibers, multiple DGC components were shown to interact with different effectors to regulate intracellular signalling cascades.^21^ For instance, calmodulin was shown to bind to dystrophin and syntrophin to regulate the Akt pathway, while the growth factor receptor-bound protein 2 (Grb2) binds to β-Dag and activates the mitogen-activated protein kinases (MAPK) pathway.^22^ Microarray data comparing prospectively isolated satellite cells to differentiating myotubes showed that multiple components of the DGC such as dystrophin, syntrophin, Dag1 and sarcoglycan are expressed in satellite cells as well as in myofibers.^18^ Therefore, in addition to its role in the regulation of Mark2 activity, dystrophin and other members of the DGC could potentially activate various signalling cascades regulating satellite cell behaviour (Figure 2).

In myofibers, binding of Dag1 to laminin was shown to cause the recruitment of Rac1 to syntrophin.^23^ The small GTPase Rac1 is involved in actin cytoskeleton rearrangement and it is known to interact with the PAR complex to establish mitotic spindle orientation and asymmetric cell division.^24^ Consistent with the hypothesis that the DGC–Rac1 interaction regulates satellite cell behaviour, it was shown in C2C12 myoblasts that exposure to laminin recruits Rac1 to the DGC, which activates the JNK signalling pathway and increases cell proliferation.^23^ Another effector, the neuronal nitric oxide synthase (nNOS), was shown to bind to syntrophin and regulate nitric oxide (NO) production in myofibers.^21,25^ Notably, NO has been demonstrated to have a direct effect on satellite cell proliferation and self-renewal through a Vangl2-dependent noncanonical Wnt pathway.^26^ NO delivery was also shown to partially rescue satellite cell dysfunction in α-sarcoglycan-null dystrophic mice and to promote muscle regeneration.^26^ Altogether, these findings suggest that DGC components expressed by the satellite cells interact with multiple signalling effectors and could be involved in the regulation of satellite cell behaviour through additional mechanisms other than Mark2-dependent cell polarity establishment (Figure 2). Understanding how other members of the DGC regulate signalling pathways in satellite cells and whether these functions are dysregulated in DMD could help to develop a range of therapeutic compounds that rescue dysfunctions in *DMD*-mutant satellite cells.

## Therapeutic avenues

Over the last decades, multiple therapeutic avenues have been explored to treat Duchenne muscular dystrophy, such as cell therapies,^27^ gene therapies and other pharmacological compounds. Here we discuss therapies that could be use to target cell-autonomous defects in *DMD*-mutant satellite cells.

### Gene therapy

Many therapies aiming to restore dystrophin expression such as exon skipping or gene delivery are currently in the preclinical or early clinical phase.^28^ Despite their great therapeutic potential, gene therapies have led so far to mild dystrophin re-expression in DMD muscles and poor improvement in muscle phenotype owing to multiple technical issues such as lack of specificity, poor delivery and low efficiency. Recently, studies from different groups showed that adeno-associated viruses (AAV) delivery of CRISPR-Cas9 endonucleases coupled with paired guided RNAs can be used to remove the mutated exon 23 in *mdx* mice and restore *Dmd* expression in postnatal muscles.^29–31^ Depending on the delivery method (systemic or local), the level of dystrophin restoration is mild to moderate, but, interestingly, this technique was also shown to restore dystrophin expression into satellite cells, albeit to a lower level.^29^ AAV gene delivery is inefficient into quiescent satellite cells but its efficiency is increased into proliferating myoblasts.^32^ Further studies are needed to improve AAV transduction efficiency into satellite cells and, in this regard, the recent identification of the AAV receptor is a step forward in the development of new tools to improve AAV efficacy.^33^ Moreover, even with limited efficiency, it was showed that lentiviral-mediated gene delivery of micro-dystrophin into a small proportion of satellite cells can sustain dystrophin expression in myofibers for years.^34^ Therefore, satellite cells are a primary target to improve long-term efficacy of gene therapy due to their high self-renewal capacity.

In addition to improve long-term engraftment, gene therapies designed to rescue the intrinsic defects of satellite cells could be an alternative approach. For instance, in *fukutin*-deficient dystrophic mice, administration of an AAV vector containing the *fukutin* complementary DNA under the CMV promoter (leading to ubiquitous gene expression) leads to better muscle regeneration compared with the muscle creatine kinase promoter (myofiber specific), indicating that the restoration of fukutin expression in satellite cells improve their regenerative potential.^17^ However, restoring dystrophin expression in DMD is challenging considering the large size of the *DMD* gene. To circumvent this technical challenge, micro-dystrophin complementary DNA was created to fit into AAV vectors.^35^ Micro-dystrophin lacks most of the *DMD* rod domain and generates a truncated dystrophin protein that does not contain the Mark2-binding site (spectrin-like repeats R8–R9 corresponding to exon 26–30 of the *DMD* gene). These micro-dystrophin proteins are able to functionally restore myofibers stability, however, they cannot rescue the polarity defect in dystrophin-deficient satellite cells. Consistent with the important role of the *DMD* domain containing the Mark2-binding site (exon 26–30), analysis of the severity of Becker muscular dystrophy phenotype shows that mutations in the proximal rod domain of *DMD* (exon 10–32) causes a more severe muscle phenotype compared to mutations in the central rod domain (exon 33–45).^36,37^ Therefore, it may be beneficial for the development of micro-dystrophin vectors to include the Mark2 binding site in order to rescue satellite cell intrinsic defects and improving long-term efficacy.

### Pharmacological drugs

**Figure 3:**
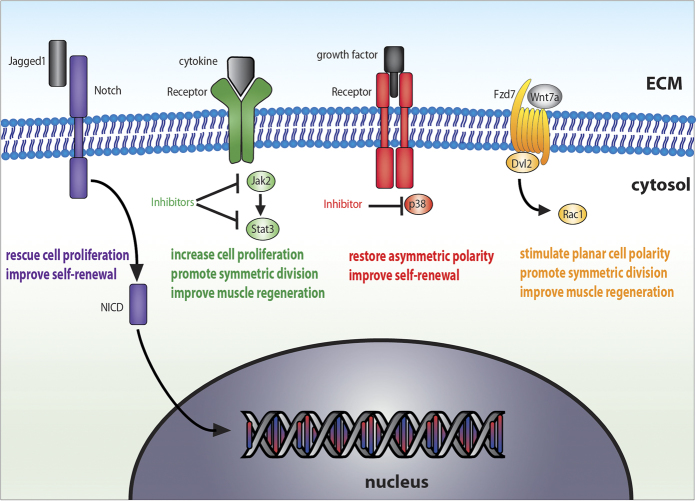
Potential therapeutic targets to improve dystrophin-deficient satellite cell function. Schematic micrograph of a satellite cell membrane. Various signalling pathways are dysregulated in dystrophin-deficient satellite cells. Different therapeutic compounds such as Wnt7a, Notch activators, Jak2/Stat3 inhibitors and p38MAPK inhibitors were shown to rescue satellite cell function and are potential therapeutic targets.

The finding that dystrophin-deficient satellite cells are intrinsically defective suggest that therapies enhancing satellite cell myogenic capacity have the potential to improve muscle regeneration and ameliorate the quality of life of DMD patients (Figure 3). Consistent with this hypothesis, overexpression of Jagged1, a Notch ligand, was shown to improve muscle regeneration and function in dystrophic dogs, a mechanism that is partially mediated by increased myogenic cell proliferation.^38^ Recent case studies also showed that some DMD patients have the remarkable capacity to maintain their muscle function and walking ability despite the complete absence of dystrophin, however it remains unknown whether this effect is caused by overexpression in signalling pathways, such as Notch, that enhance satellite cell function.^39,40^ The Notch pathway is also involved in asymmetric cell division as demonstrated by the asymmetric inheritance of Notch ligand Delta-1 and the Notch interacting protein Numb in the committed daughter cell following asymmetric satellite cell division, but it remains to be determined whether Notch overexpression rescues the asymmetric polarity deficit observed in dystrophin-deficient satellite cells.^19,41^ Consistent with the positive role of Notch/Numb on satellite cell function, it was shown that inactivation of Numb in *mdx* mice promotes senescence of myogenic cells and exacerbates the dystrophic muscle phenotype.^42^ Therefore, the Notch pathway is an interesting target to improve satellite cell function, however, constitutive activation of the Notch pathway specifically in *mdx* satellite cells through Notch intracellular domain overexpression was shown to restore satellite cell self-renewal, but it did not translate into better regenerative potential.^6^ This discrepancy can be explained by the fact that overexpression of Notch pathway promotes self-renewal, but represses satellite cell myogenic progression, a process that is already insufficient in *mdx* satellite cells.^18^ Therefore, Notch is a potential therapeutic target to rescue satellite cell proliferation and self-renewal, however, it needs to be transiently activated and/or used in combination with other compounds promoting myogenic progression.

Various other signalling pathways involved in cell polarity establishment were also shown to be dysregulated in pathological conditions and could be targeted to rescue satellite cell functions (Figure 3). For instance, the Jak/Stat signalling pathway regulates asymmetric cell division and the generation of myogenic progenitors, and its activity is chronically upregulated in satellite cell during aging.^43,44^ Notably, the use of Stat3 inhibitors partially rescues satellite cell proliferation and muscle regeneration in dystrophic muscles.^44^ Another signalling pathway, the p38MAPK pathway was shown to be involved in the regulation of satellite cell asymmetric division.^45^ In aged muscles, p38MAPK signalling pathway is chronically upregulated in satellite cells and p38MAPK inhibitor was shown to partially restore the establishment of asymmetric cell polarity and improve self-renewal of satellite cells.^46^ p38MAPK is also aberrantly upregulated in regenerating *mdx* muscle and pharmacological inhibition of p38MAPK in dystrophic mice was shown to improve muscle phenotype.^47,48^ However, the exact effect of p38MAPK inhibitors on dystrophin-deficient satellite cells remains to be determined.

The potential of biological molecules acting on satellite cell polarity should be evaluated as therapeutic approaches to restore dystrophin-deficient satellite cell function (Figure 3). For instance, Wnt7a is known to stimulate the planar cell polarity pathway through its interaction with Fzd7 receptor, which promotes satellite stem cell expansion through symmetric cell division.^49,50^ Noticeably, Wnt7a injection into the TA muscle of *mdx* mice was shown to increase the number of activated satellite cells (Pax7+/MyoD+), myofiber size and muscle force.^51^ Notably, a truncated variant containing only the C-terminal portion of Wnt7a was shown to retain its full biological activity and has enhanced dispersion compared with full-length Wnt7a, which is highly relevant for the development of therapeutic drugs.^52^ Another factor, the granulocyte-colony stimulating factor (G-CSF) receptor is asymmetrically segregated during asymmetric satellite cell division and treatment with G-CSF was shown to improve muscle regeneration in dystrophic mice.^53^ Altogether, these results suggest that alternative mechanisms exist to restore satellite cell function with the lack of dystrophin. However, whether the positive effects of Wnt7a or G-CSF on dystrophic muscle regeneration are mediated by the reestablishment of satellite cell polarity remained to be determined.

Various pharmaceutical components have the potential to improve the myogenic potential of dystrophin-deficient satellite cells by rescuing asymmetric division or by promoting alternate cell polarity establishment that rescues aberrant cell division. Many of these drugs are already approved for the treatment of different diseases, however, systemic delivery of these compounds is likely to have off-target effects and a cost-benefits analysis should be assessed. Moreover, some signalling pathways affected by these drugs are also important for myogenic progression and should be only transiently targeted. Nonetheless, these new findings indicate that satellite cells are an important therapeutic target that could improve muscle regeneration and preserve muscle function over time in DMD patients (Figure 3).

## Implications on various diseases

### Other muscular dystrophies

Muscular dystrophies (MDs) are a group of heterogeneous diseases (>30 different types of MDs) that are characterised by muscle weakness and dysfunction, but that are caused by different mutations in genes that encode for various proteins. Many mutations affect genes that encode for proteins interacting, directly or indirectly, with the DGC, such as Dag1, apha7-integrin (itga7), fukutin, Large, laminin and so on.^54^ Similar to what was observed in dystrophin-deficient satellite cells, these mutations could lead to intrinsic defects in satellite cell behavior. For instance, deletion of *Dag1* in satellite cells was previously suggested to impair their regenerative potential.^16^ Conditional deletion of *fukutin*, which is important for Dag1 glycosylation and function, under the control of the Myf5 promoter was demonstrated to impair satellite cell proliferation, differentiation and skeletal muscle regeneration.^17,55^ Likewise, deletion of *Large*, which encodes for another glycosyltransferase that glycosylates Dag1, was shown to impair satellite cell proliferation *in situ*, however, when the satellite cells are removed from their niche and cultured *in vitro* the proliferation capacity is restored.^56^ A similar inconsistency between *in situ* and *in vitro* results was also observed in *mdx* satellite cells, where the decrease in satellite cell proliferation observed on isolated myofiber culture is not detectable when primary myoblasts are cultured out of their niche in a dish.^18^ These results suggests that the interaction of the DGC with its microenvironment is important *in situ* for cell polarity establishment and cell division, however, this interaction is not needed for cell division *in vitro* where there is a lack of polarity cues from the microenvironment. This hypothesis is supported by the fact that the expression of the DGC components is loss when primary myoblasts are cultured *in vitro*^18^ together with their ability to perform asymmetric cell division.^57^

The DGC complex was also shown by proximity ligation assay to physically interact in myofibers and in satellite cells with itga7, another laminin-binding protein.^18^ Muscle deficient in itga7 shows classical symptoms muscular dystrophy.^58^ Double knockout of itga7 and dystrophin/Dag1 strongly increases the regeneration deficit suggesting that these two laminin-binding proteins have complementary roles.^59^ Similar to dystrophin-deficient satellite cells, absence of itga7 leads to a strong reduction in satellite cell proliferation and differentiation.^60^ Whether integrins regulates cell polarity establishment in satellite cell as it does in other stem cell types remained to be determined.^61^

Utrophin is a gene homologous to the dystrophin gene (although considerably shorter in length) with highly conserved sequence at the N-terminal and C-terminal ends, but with less similarities at the rod domain.^62^ Utrophin is widely expressed in various tissues, but its expression in healthy adult skeletal muscle is restricted at the neuromuscular and myotendinous junctions. In dystrophin-deficient muscles, utrophin expression is also detectable in the myofibers; and mice deficient for both dystrophin and utrophin display worst muscle phenotype, suggesting that these two proteins have complementary functions.^63,64^ Furthermore, overexpression of utrophin in *mdx* mice reduces the dystrophic phenotype.^65,66^ It is unknown whether utrophin is expressed in satellite cells, however, it would be interesting to determine since utrophin also possesses the binding site for Mark2.^67^ Notably, it was shown that satellite cells from mice double knockout for dystrophin and utrophin exhibit proliferation and differentiation defects stronger than in *mdx* mice, however, it is unclear whether this defect is cell autonomous.^68^

Altogether, these findings indicate that interaction of satellite cells with their microenvironment provides critical feedbacks for the establishment of cell polarity and mutations in genes that encode for proteins of the DGC do not only lead to myofiber fragility, but also to intrinsic defects in satellite cells in a variety of MDs.

### Stem cells in other tissues

The *DMD* gene encodes for different isoforms of dystrophin generated by different promoters or by alternative splicing. These different dystrophin protein (Dp) isoforms are classified based on their size in kDa, i.e., Dp427, Dp260, Dp140, Dp116, Dp71 and Dp40. Some isoforms of dystrophin are ubiquitous such as Dp71, whereas others are tissue-specific such as Dp427 (skeletal muscle, brain and heart), Dp260 (retina, brain and heart), Dp140 (central nervous system and kidney) and Dp116 (Schwann cells). The point mutation in *mdx* mice is located at exon 23 leading to the lack of full-length dystrophin (Dp427) but it does not affect the other isoforms.^69–71^

Expression of dystrophin in various tissues suggests that it could regulate polarity establishment and asymmetric division in stem cells from other tissues. For instance, expression of Dag1 was shown to be important to drive cell polarity establishment in epithelial cells.^72^ Impaired stem cell function could be partially responsible for other symptoms observed in DMD patients. For example, DMD patients and *mdx* mice have higher prevalence of cognitive impairment with disorganised central nervous system architecture and reduced number of neurons, a phenotype that is worsen by the absence of the Dp140 isoform.^73,74^ Brain-specific deletion of *Dag1* using various Cre drivers leads to brain abnormality partially caused by reduced laminin-binding ability and neuronal migration errors.^75^ Furthermore, asymmetric division of neuroblasts is critical to generate the ganglion mother cell that will give rise to neurons and it could be hypothesised that dystrophin/DGC has a role to play in the regulation of neuroblast asymmetric cell division that is similar to what is observed in satellite cells. Therefore, intrinsic defects in polarity establishment and asymmetric cell division should be evaluated in stem cells from tissues other than skeletal muscles such as brain and retina.

### Rhabdomyosarcoma

Mutations in the *DMD* gene have also been associated with higher incidence of various mesenchymal tumours, such as gastrointestinal stromal tumour, rhabdomyosarcoma and leiomyosarcoma.^76,77^ Dystrophin re-expression with mini-dystrophin vector reduced the invasiveness and the migration of myogenic sarcoma cells, which demonstrates that dystrophin has a tumour suppressive effect.^76^ Notably, mutations associated with myogenic cancers lead to deletion of the Dp427 dystrophin protein but preserved the Dp71 isoform, suggesting that Dp71 supports tumour formation and that expression of full-length dystrophin is required to preserve the tumour-suppressive effect.^76^ Consistent with these results, our findings indicate that absence of full-length dystrophin in satellite cells impairs asymmetric cell division,^18^ which has been related to formation of tumours in different tissues.^78^ Dystrophin-deficient satellite cells also display abnormal division patterns, which is consistent with previous studies showing that myoblasts isolated from *mdx* mice or dystrophic patients and cultured *in vitro* have higher proportion of cancer-like aberrations such as somatic aneuploidy and DNA damage response.^77^ Altogether, these results suggest that regulation of cell polarity by dystrophin is a common mechanism that is dysregulated both in DMD and in myogenic tumour.

## Conclusion

The findings that dystrophin-deficient satellite cells are intrinsically defective in DMD patient indicate that they could be targeted by therapeutic compounds. Drugs aiming to restore polarity and proper mitotic division could be used as part of a global therapy for DMD, which could include other treatments such as anti-inflammatory and anti-fibrosis drugs.^79^ A combined therapeutic approach that improves satellite cell function in DMD patients could transform this severely debilitating and lethal disease into a manageable chronic condition. Moreover, owing to their high self-renewal capacity, rescuing satellite cell dysfunction could also be beneficial for gene therapies and increase their long-term efficacy.
